# Evolution of the Properties and Composition of Heavy Oil by Injecting Dry Boiler Flue Gas

**DOI:** 10.1021/acsomega.1c05101

**Published:** 2021-12-12

**Authors:** Wenjie Dong, Yanmin Ji, Jian Wang, Baogang Li, Farong Yang, Jijin Yang

**Affiliations:** † Key Laboratory of Shale Gas and Geoengineering, Institute of Geology and Geophysics, Chinese Academy of Sciences, Beijing 100029, China; ‡ College of Earth and Planetary Sciences, University of Chinese Academy of Sciences, Beijing 100029, China; § Innovation Academy for Earth Science, Chinese Academy of Sciences, Beijing 100029, China; ∥ Lusheng Petroleum Development Co. Ltd., SINOPEC Shengli Oil Field, Dongying, Shandong 257077, China; ⊥ Bohai Central Lab, Engineering Technology Branch, CNOOC Energy Development Co. Ltd., Tianjin 300452, China

## Abstract

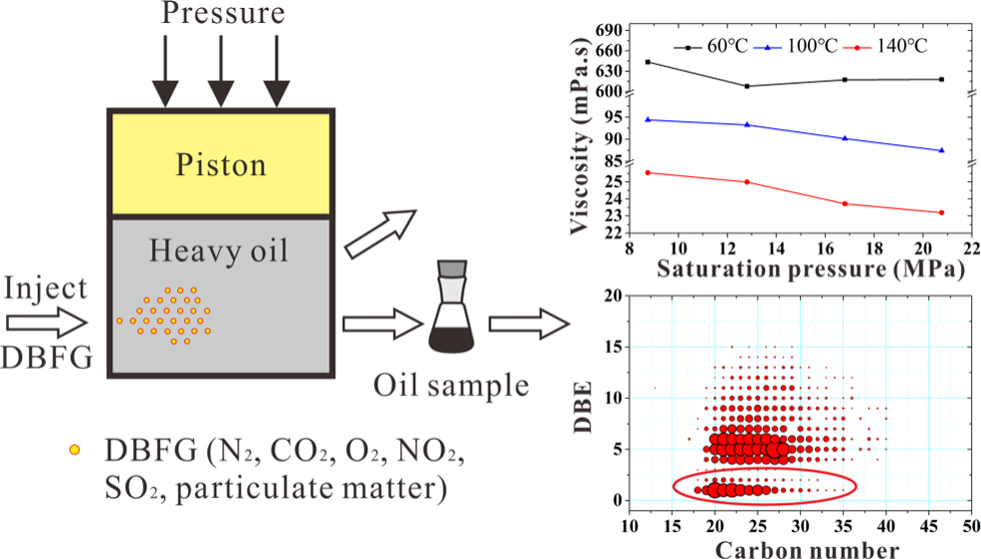

As the increasing pressure to carbon peak and carbon neutral has brought carbon capture and storage (CCS) to the forefront as an emission mitigation tool, greater attention is being paid to the potential for injecting dry boiler flue gas (DBFG) into oil reservoirs. With the aim to directly inject DBFG with steam into heavy oil reservoirs, this study presents the results of a laboratory investigation of the effect of DBFG on the properties and composition of heavy oil by viscosity measurement, pressure–volume–temperature measurement, high-temperature and high-pressure experiment, and high-resolution mass spectrometry analysis. The results of the experiments show that adding 0.5 wt % particulate matter has no obvious influence on the viscosity of heavy oil. DBFG dissolved in heavy oil can reduce viscosity, increase the flow capability, and make the heavy oil volume swell. Heavy oil is oxidized with DBFG at 140 °C, which is mainly caused by the O_2_ in the DBFG, and the oxidation product is alcohol. The findings of the beneficial effect of DBFG on viscosity and swelling factor and the negligible negative effect of the small amount of nitrogen oxides, sulfides, and particulate matter in DBFG are very encouraging. It is expected that DBFG can be directly injected into heavy oil, not only for enhanced oil recovery (EOR) but also for reducing the emissions of greenhouse gases and pollutants, as well as for saving costs.

## 1 Introduction

China is the world’s largest emitter of greenhouse gases and emitted 10.2 billion tons of CO_2_ in 2019.^1^ Eighty-six percent of carbon sources come from the utilization of fossil fuels, i.e., coal, oil, and natural gas.^2^ To fight against climate change, China aims to be carbon peak before 2030 and carbon neutral by 2060 through cutting greenhouse gases. CO_2_-EOR (enhanced oil recovery), as one CO_2_ utilization pathway, is suitable for more than 90% of the world’s oil reservoirs and can store as much as 140 billion tons of CO_2_.^3−6^

CO_2_-EOR, together with N_2_-EOR, is widely used as a gas injection technique to change the physical and chemical properties of heavy oil, such as viscosity and interfacial tension.^7,8^ High-temperature steam, as a thermal injection technique, can also be very effective in reducing the viscosity of crude oil in a reservoir.^9^ However, there is a price to pay for using pure CO_2_, N_2_, or steam as enhanced oil recovery (EOR) techniques in the oil field. Oil production companies have to either buy pure CO_2_ or N_2_ from other gas manufacturers or generate it by themselves, and sometimes transportation is required if the gases are not commercially available locally. Steam is typically generated locally by the burning of fossil fuels in the boiler. One big disadvantage of steam generation is its byproduct, i.e., boiler flue gas (BFG), a waste gas produced by the burning of fossil fuels. Taking the Shengli Oilfield as an example, 80 × 10^8^ m^3^ of BFG is emitted each year while generating steam. The main compositions of BFG are N_2_, CO_2_, O_2_, water vapor, and small amounts of nitrogen oxides, sulfur compounds, and particulate matter, and its temperature can be several hundred degrees in Celsius before cooling.^10,11^ If the BFG experiences the cooling and dewatering process, it becomes DBFG. The components nitrogen oxides, sulfur compounds, particulate matter, and CO_2_ in the BFG from the steam generator in the oil field are either stable long-term pollution gases and particulate sources or become greenhouse gases if released to the atmosphere without collection and treatment.^12,13^ One example is that particulate matter may be covered with toxic or carcinogenic substances and can prove harmful for human beings.^11−13^ Since DBFG has a high temperature and clearly contains several components, i.e., CO_2_, N_2_, O_2_, and so on, it is very interesting to see whether we can take advantage of them in the EOR process. In addition, stopping the emission of greenhouse gas and particulate matter to the atmosphere could prove more important to human history in the long term by taking advantage of these components.

Laboratory research^8,14−16^ and field applications^17,18^ have shown that CO_2_ and N_2_ are two beneficial components for heavy oil EOR. A series of pressure–volume–temperature (PVT) experiments were conducted to study the solubility of CO_2_, N_2_, and their mixtures in heavy oil and their effect on swelling, density, and viscosity reduction of heavy oil.^8,15,19−21^ These investigations showed that CO_2_ has a high solubility in heavy oil and can reduce the viscosity of heavy oil and make it appear swollen.^15,19,20,22,23^ The solubility of N_2_ in heavy oil is less than that of CO_2_.^19,24,25^ Under the same pressure condition, the solubility of N_2_ in heavy oil increases with increasing temperature,^24^ while other studies showed the opposite result.^19^ The mixture of CO_2_ and N_2_ has a similar effect to CO_2_ but to a lesser degree.^8,20,21,26^

Although O_2_ is a reactive element, the presence of 5 mol % O_2_ in a gas mixture at room temperature has a negligible effect on the PVT properties of the heavy oil–flue gas system.^21^ As the gas-assisted steam method for heavy oil recovery can reach 100–300 °C, it is unknown whether and how O_2_ at this temperature range will affect the properties and composition of the heavy oil when steam and O_2_ are present. The effect of nitrogen oxides, sulfur compounds, and particulate matter in DBFG on the properties and composition of heavy oil is unknown. In consideration of the serious influence of heavy oil properties and composition alteration on the EOR process, it is important to study the evolution of properties and composition of heavy oil by injecting DBFG.

In this paper, with the aim to see the possibility of directly injecting high-temperature DBFG with the main components, i.e., CO_2_, N_2_, O_2_, and small amounts of nitrogen oxides, sulfur compounds, and particulate matter, into heavy oil during the EOR process, we carried out a series of experiments and were interested in understanding how O_2_, nitrogen oxides, sulfur compounds, and particulate matter affect the heavy oil properties and composition at the evaluated temperature. First, the morphological structure, particle size distribution, and elemental composition of particulate matter from DBFG and the influence of particulate matter on the viscosity of heavy oil were analyzed. Second, a PVT experiment was conducted to understand the solubility of DBFG and its effect on swelling, viscosity reduction, and density of the heavy oil. Third, high-temperature and high-pressure (HTHP) experiment and high-resolution mass spectrometry analysis were performed to study the influence of small amounts of nitrogen oxides, sulfur compounds, and O_2_ in DBFG on heavy oil composition.

## 2 Results and Discussion

### 2.1 Particulate Matter

#### 2.1.1 Microscopic Morphology of Particulate Matter

**Figure 1:**
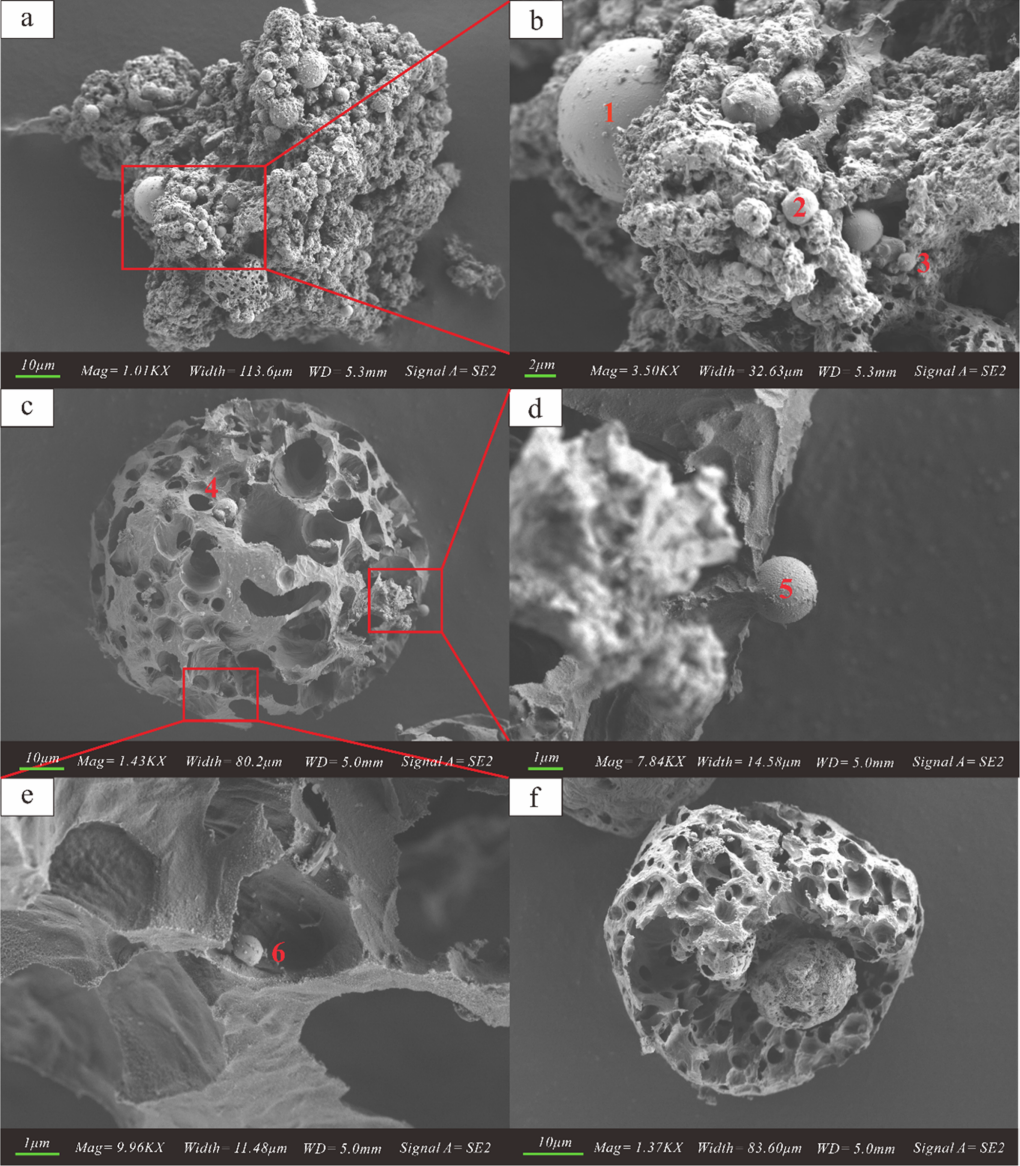
SEM images of particulate matter: (a) particulate matter aggregates; (b) dense particles 1–3 with diameters of 10.48, 2.294, and 1.083 μm; (c) cenosphere and dense particle 4 with a diameter of 3.446 μm, (d) dense particle 5 with a diameter of 1.722 μm, (e) dense particle 6 deposited on cenospheres with a diameter of 694 nm, and (f) ruptured cenospheres.

**Figure 2:**
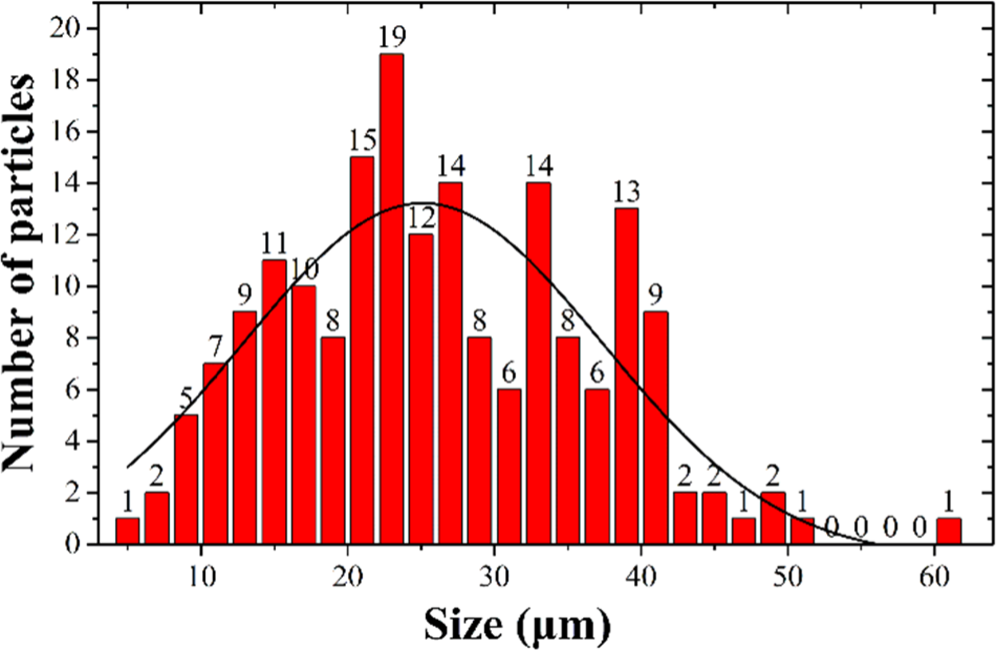
PSD of the single cenosphere particulate matter.

**Figure 3:**
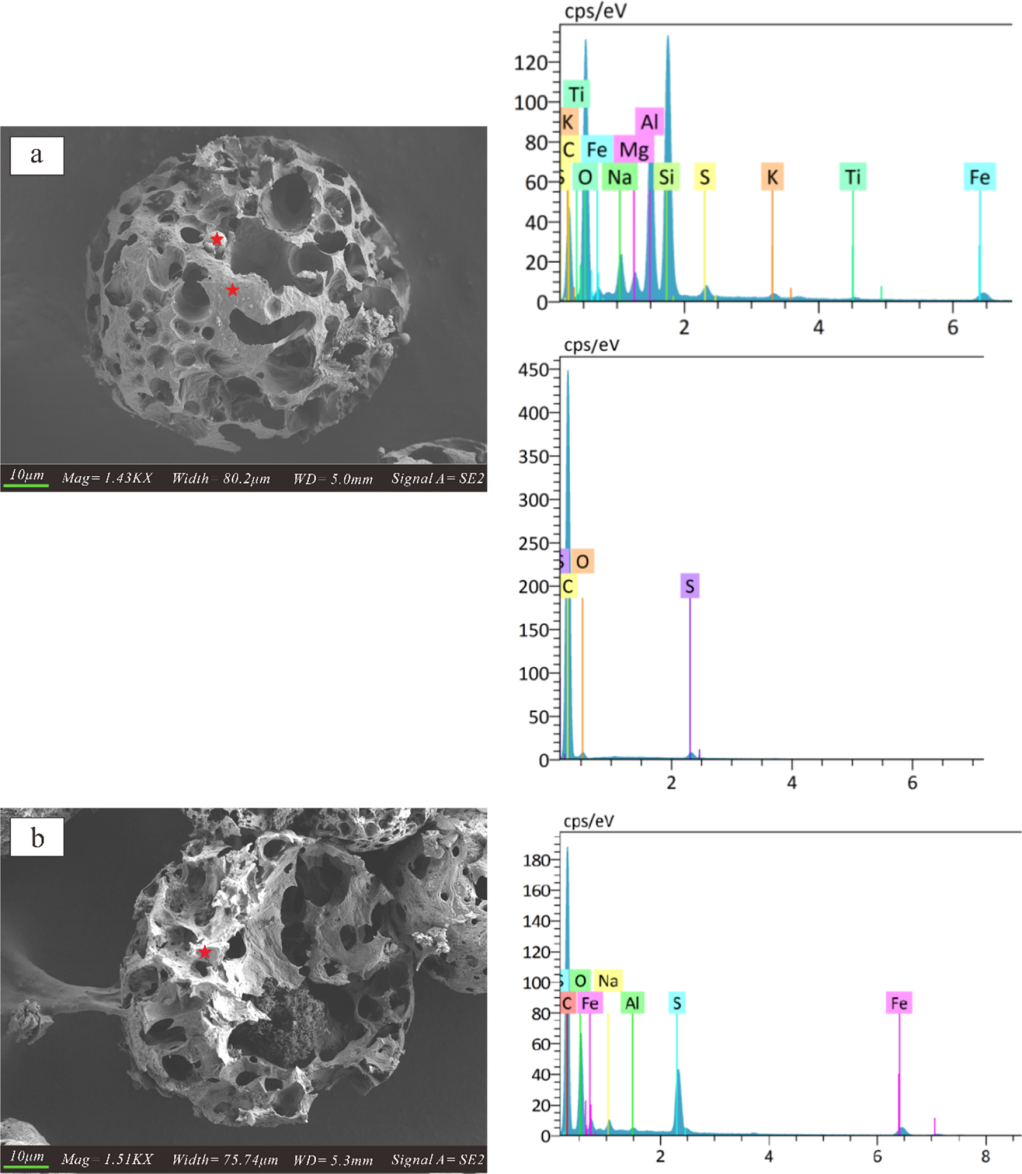
SEM-EDS analysis: (a) on the dense particle surface and on the surface of cenospheres, and (b) inside the cenospheres.

Morphologically, the fly ash particulate matter from burning heavy oil shows two different structures: cenospheres, i.e., spongy or hollow spheroid particles with a diameter of 4–62 μm, and dense particles, with a smaller diameter. Cenospheres and dense particles may mix together to form messy aggregates as shown in Figure 1, which is consistent with earlier studies.^27,28^ We sieved the solid particulate matter with 60, 150, and 300 sifts, dipped some particulate matter in a toothpick, and put them on a carbon adhesive tape; then, the particle size distribution of 187 particles was calculated by analyzing scanning electron microscope (SEM) images. The main particle size distribution (PSD) of the single cenosphere particulate matter is 10–40 μm, as shown in Figure 2. The main elements of two different structures of particulate matter are different. For example, the major elements of cenospheres are carbon, oxygen, and sulfur (Figure 3a,b), while the dense particles deposited on larger cenospheres are composed of oxygen, carbon, silicon, aluminum, and ferrum (Figure 3a). Quantitative analysis of detected elements on the surface of dense particles and cenospheres is presented in Table 1.

**Table 1:** Quantitative Analysis of Detected Elements on the Surface of Dense Particles and Cenospheres^a^

point	element	C (wt %)	norm. C (wt %)	Atom. C (atom %)	error (wt %)
a_1_	O	46.1	41.57	43.16	5.14
	C	32.02	28.88	39.94	3.83
	Si	14.54	13.11	7.76	0.63
	Al	8.8	7.94	4.89	0.43
	Fe	3.62	3.26	0.97	0.14
	Na	2.78	2.51	1.81	0.2
	Mg	1.14	1.03	0.71	0.09
	S	0.92	0.83	0.43	0.06
	K	0.5	0.45	0.19	0.04
	Ti	0.45	0.42	0.14	0.04
a_2_	C	92.64	92.64	94.66	9.67
	O	6.56	6.56	5.03	0.87
	S	0.8	0.8	0.31	0.05
b_1_	C	60.09	60.09	69.75	6.49
	O	30.63	30.63	26.7	3.47
	S	4.23	4.23	1.84	0.18
	Fe	3.74	3.74	0.93	0.14
	Na	1.1	1.1	0.67	0.1
	Al	0.2	0.2	0.1	0.04

a a_1_ is a dense particle surface; a_2_ is the surface of cenospheres; b_1_ is the surface of cenospheres.

#### 2.1.2 Influence of Particulate Matter on the Viscosity of Heavy Oil

Considering the fact that solid particulate matter is difficult to be injected into heavy oil together with DBFG in the PVT experiment, we directly added sieved particulate matter into heavy oil to understand whether particulate matter has a significant impact on the physical properties of heavy oil. Based on the emission factor for uncontrolled commercial boilers burning coal and oil, the concentration range of soot particulate matter in the BFG was calculated to be from 0.2 to 20 g/m^3^.^10,12,29^ According to the ratio of dissolved flue gas obtained in previous PVT experiments, and considering the need to add excessive DBFG in the PVT experiment in this study, we added 0.5 wt % particulate matter into heavy oil. The influence of 0.5 wt % particulate matter on the density and volume of heavy oil can be ignored through calculation. To analyze the influence of 0.5 wt % particulate matter from DBFG on the viscosity of heavy oil at different temperatures, a set of comparative viscosity measurements was conducted, and the viscosity change rate can be defined by eq 1.
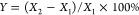
1where *Y* is the viscosity reduction rate, *X*_1_ is the original viscosity of the heavy oil (mPa.s), and *X*_2_ is the viscosity of the heavy oil dissolved with particulate matter (mPa.s).

**Figure 4:**
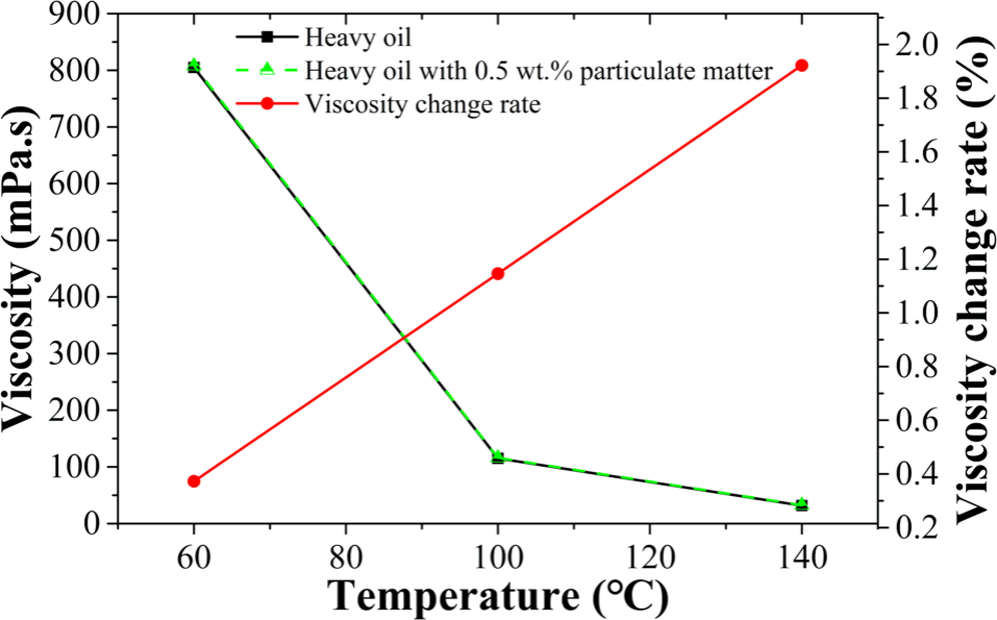
Viscosity–temperature curve of heavy oil with 0.5 wt % particulate matter.

The relationship between viscosity and temperature of heavy oil and heavy oil with 0.5 wt % particulate matter is shown in Figure 4. The result shows that adding 0.5 wt % particulate matter has no obvious influence on the viscosity of heavy oil.

### 2.2 PVT Experiment

We have proved that adding 0.5 wt % particulate matter has a negligible influence on the physical properties of heavy oil as shown in Figure 6. Thus, here, we added 0.5 wt % particular matter to dehydration dead oil in the PVT experiment to understand the solubility of DBFG and its effect on swelling, viscosity reduction, and density of heavy oil. PVT properties for mixtures of the heavy oil with DBFG are presented in Table 2.

**Table 2:** Mixtures of the Heavy Oil with DBFG at Experimental Temperature and Pressure

temperature (°C)	saturation pressure	N_2_	CO_2_	O_2_	C_1_–C_4_	C_5+_
	(MPa)	(mol %)	(mol %)	(mol %)	(mol %)	(mol %)
60	8.557	6.73	2.07	0.06	0	91.14
	12.403	9.15	2.18	0.03	0	88.64
	16.451	11.32	2.26	0.01	0.02	86.39
	20.649	13.92	2.39	0.01	0.02	83.66
100	8.829	6.72	1.22	0.06	0	92
	12.655	10.14	1.44	0.04	0	88.38
	16.672	13.75	1.65	0.04	0	84.56
	20.649	15.05	1.7	0.39^a^	0	82.86
140	8.749	9.16	1.87	0.03	0	88.94
	12.806	12.38	1.99	0.02	0	85.61
	16.803	13.75	1.65	0.04	0	84.56
	20.76	17.87	2.29	0.07	0	79.77

a Oxygen content is unexpectedly higher than the other values. This is caused by accidentally mixing with air when collecting gas for chromatography.

#### 2.2.1 DBFG Solubility

**Figure 5:**
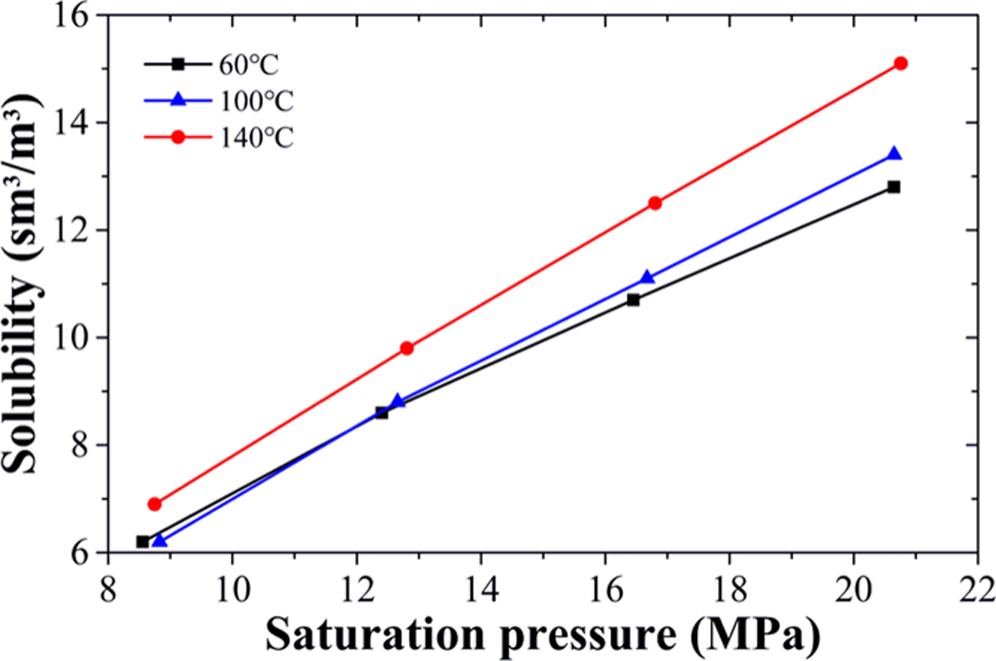
Solubility of DBFG in heavy oil under different pressure and temperature conditions.

The relationship between solubility of DBFG in heavy oil and saturation pressure under different temperatures is shown in Figure 5. The experimental results show that the solubility of DBFG increases with the saturation pressure under the same temperature. When the saturation pressure increases from 8.5 to 20.6 MPa at 60 °C, the solubility increases from 6.2 to 12.8 sm^3^/m^3^. At a similar saturation pressure range, the solubility increases from 6.9 to 15.1 sm^3^/m^3^ at 140 °C. On the other hand, under a constant pressure, the solubility increases as the temperature increases, especially at pressures above 13 MPa. This result is contrary to previous experiments.^8,15,19^ The reason for this phenomenon may be that as pressure increases, the liquid becomes much denser at lower temperatures, and molecules in the liquid phase are packed more tightly, thus leaving less room for DBFG molecules to enter.^23^ Therefore, at high pressure, the solubility of DBFG in heavy oil may increase with temperature because of the decrease in liquid density. Previous works have demonstrated that the relationship between N_2_ solubility and temperature is completely opposite to that between CO_2_ solubility and temperature in extra-heavy oil, i.e., as the temperature increased, the solubility of the N_2_ in extra-heavy oil increased.^24^ The increase becomes more obvious under HTHP conditions. Another possible reason for this is that DBFG and heavy oil have undergone complex chemical reactions, which cause a change in the composition of heavy oil and then could dissolve more DBFG. Further work is required to explain this phenomenon.

#### 2.2.2 Swelling Factor for Heavy Oil Saturated with DBFG

The swelling factor was calculated from
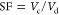
2 where SF is the swelling factor, *V*_c_ is the volume of heavy oil saturated with DBFG at the temperature and pressure of the system, and *V*_d_ is the volume of dead oil in the cell at the temperature of the system.^30^

**Figure 6:**
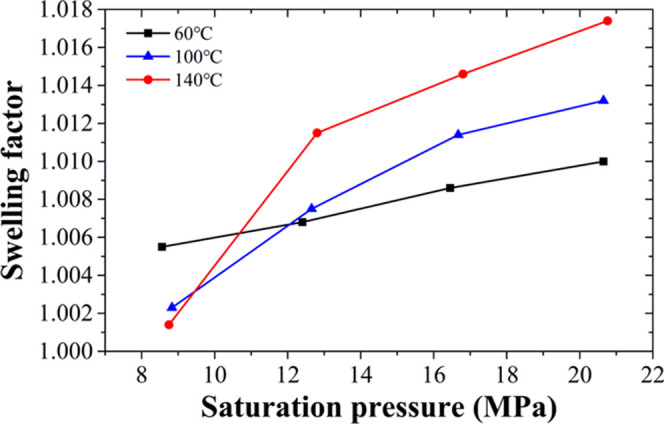
Swelling factor of heavy oil saturated with DBFG under different pressure and temperature conditions.

The relationship between saturation pressure and swelling factor for the heavy oil under different temperatures is presented in Figure 6. The experimental results illustrate that under the same temperature, the swelling factor of heavy oil saturated with DBFG increases as the pressure increases. When the saturation pressure is in the range of 8–13 MPa, the swelling factor reduces with an increase in temperature at the same saturation pressure, while when the saturation pressure is above 13 MPa, the situation is reversed, the swelling factor increases with the increase in temperature at the same saturation pressure. The maximum swelling factor value is 1.0173. The swelling factor curves have the same behavior as the curve of DBFG solubility, but different from other studies, which showed that the swelling factor reduced with the increase in temperature at the same saturation pressure.^8,15^ Whether it is caused by physical change or chemical change requires further research.

#### 2.2.3 Viscosity for Heavy Oil Saturated with DBFG

**Figure 7:**
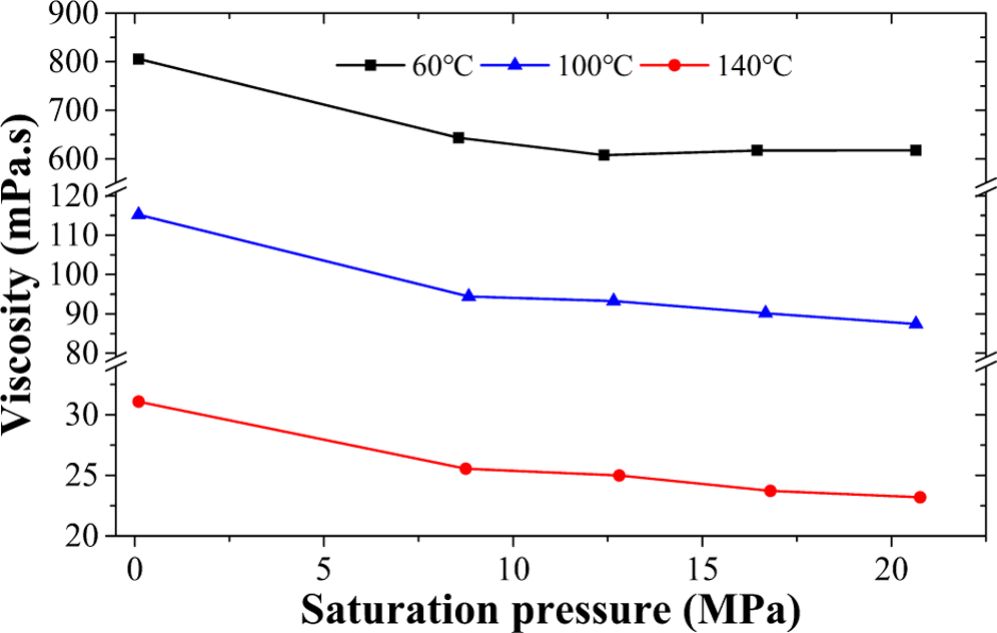
Viscosity of heavy oil saturated with DBFG under different pressure and temperature conditions.

**Figure 8:**
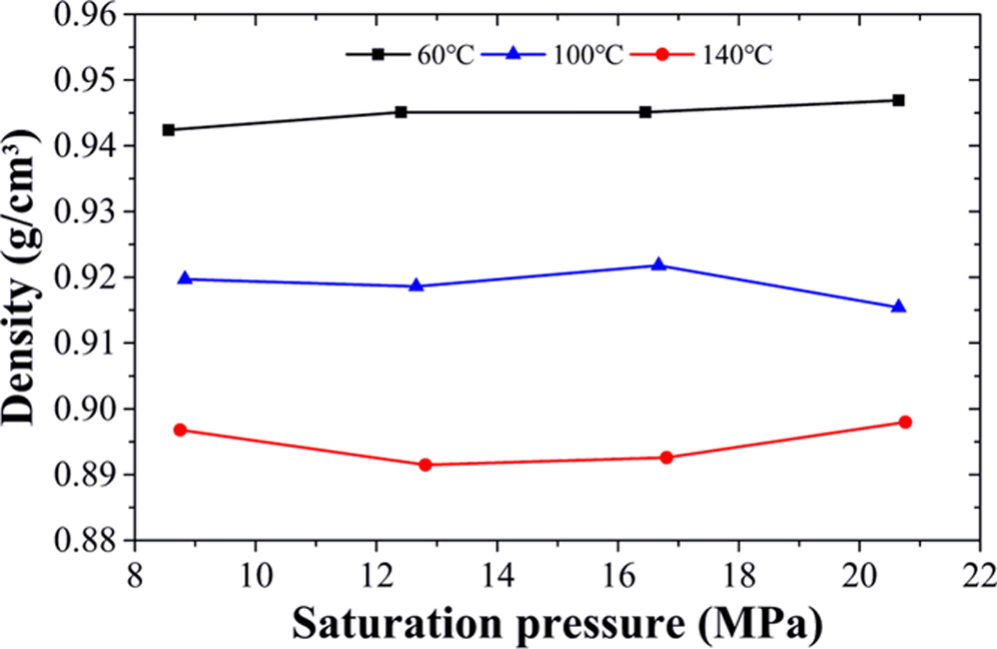
Density of heavy oil saturated with DBFG under different pressure and temperature conditions.

It has been confirmed in the last section that the influence of 0.5% soot particulate matter on the viscosity of heavy oil can be negligible. This section mainly considers the influence of DBFG on the viscosity of heavy oil. The relationship between saturation pressure and viscosity under different temperatures is shown in Figure 7. The experimental results demonstrate that under the same temperature, the viscosity reduces with increasing saturation pressure; at the same time, it also shows that DBFG can reduce the viscosity of heavy oil, especially under HTHP conditions. For example, the viscosity reduction rate increases from 20.11 to 24.55% at 60 °C, the viscosity reduction rate increases from 18.04 to 24.1% at 100 °C, and the viscosity reduction rate increases from 17.78 to 25.38% at 140 °C. The CO_2_ in the DBFG dissolves into the heavy oil, which destroys the original micelle structure of the heavy oil and greatly reduces the viscosity of the heavy oil. The viscosity curves of heavy oil saturated with DBFG at 60 °C begin to rise at high pressures after reaching a minimum because the density of the DBFG–heavy oil mixture increases at such high pressures, consistent with the density result (Figure 8).

#### 2.2.4 Density of Heavy Oil Saturated with DBFG

The relationship between saturation pressure and density under different temperatures is shown in Figure 8. The experimental results show that under the same saturation pressure condition, the density of heavy oil saturated with DBFG decreases as the temperature increases. However, under the same temperature condition, the density of heavy oil saturated with DBFG shows an insignificant increasing trend as the saturation pressure increases, perhaps as a result of the high pressure.

#### 2.2.5 Asphaltene Stability

It is well-known that the CO_2_ injected into the heavy oil destabilizes the asphaltenes, which precipitate out of the oil. During CO_2_ flooding, with the increase in concentration of CO_2_ in crude oil, the surface of the asphaltene molecule can be gradually occupied by a large number of CO_2_ with a small molecular size, so that the content of colloid solvents on asphaltene molecules is gradually decreased, and the stability of the asphaltene micelle is destroyed, leading to the precipitation of the asphaltene.^31^ Asphaltene precipitation may cause produced crude oil to be deasphalted, becoming lighter and less viscous in comparison with the original crude oil. In addition, asphaltene deposition on the rock surface may cause reservoir plugging and wettability alteration, which might eventually lead to a reduction of enhanced oil recovery performance.^32−34^ Asphaltene precipitation can also severely damage the wellbore region, plugging the production pipelines and reducing the capacity of surface facilities.^35^

A series of studies have shown that asphaltene precipitation increases with the increase in CO_2_ injection.^36,37^ During CO_2_ flooding, the asphaltene-to-resin ratio of crude oil is altered, causing asphaltene precipitation, although there is a small amount of N_2_ and CH_4_.^37^ The mechanism for CO_2_-induced asphaltene precipitation is temperature-dependent, i.e., as the temperature increases, asphaltene particles tend to flocculate and make larger particles.^35,36^

DBFG contains 9% CO_2_, and asphaltene will precipitate after DBFG comes in contact with heavy oil. This phenomenon could be critical, especially during the DBFG-assisted steam stimulation EOR method.

PVT experiments show that the DBFG dissolved in heavy oil can reduce the oil viscosity, increase the flow capability, and make the heavy oil volume swell, in which CO_2_ plays a major role. Under a constant pressure, the solubility and swelling factor increase as the temperature increases, especially when the pressure is above 13 MPa. It is of great significance to understand the mechanism of EOR in heavy oil and carbon storage at the microscale. The results of the solubility and swelling factor are contrary to those of other studies;^8,15,26^ therefore, a set of HTHP experiments and high-resolution mass spectrometry analysis has been carried out to understand this difference in the next section.

### 2.3. Influence of DBFG on Heavy Oil Components

#### 2.3.1 HTHP Reaction Experiment

**Figure 9:**
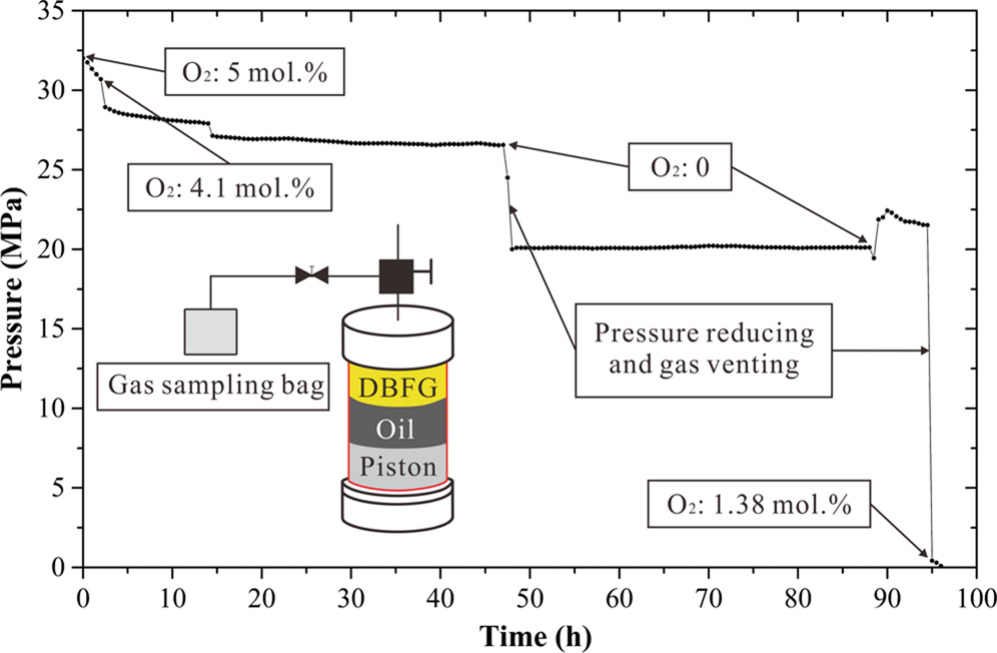
Changes in O_2_ content of the gas phase in the reaction still during the HTHP reaction experiment.

During the HTHP reaction experiment at a constant temperature of 140 °C, we measure the O_2_ content of the gas phase in the reaction still through the gas sampling method for gas chromatography analysis five times, as shown in Figure 9, in which the second measurement (at the 14th hour) failed because of mixing with air. At the 2nd, 14th, and 88th hours, we collected some gas with the gas sampling bag for chromatographic analysis, which led to a slight reduction in pressure in the reaction still. At the 47th and 94th hours, a significant pressure drop happened, which was caused by adjusting the pump and discharging the excess gas. The initial O_2_ content in the DBFG was 5 mol %. After 2 h, the gas pressure in the reaction still reduced from 32 to 30.48 MPa and the O_2_ content in the gas phase was 4.1 mol %, indicating that 0.9 mol % O_2_ was consumed either by dissolution or reaction. Then, the pressure gradually decreased until the 40th hour, and the pressure reached a steady state in 40–47 h; meanwhile, no O_2_ could be detected in the gas phase at the 47th hour, indicating that all O_2_ enters the oil phase. After the O_2_ content was measured at the 47th hour, we reduced the pressure to 20 MPa and discharged the excess gas so that the oil and gas in the reactor were in a single phase and kept the pressure constant for 40 h. At the 88th hour, the gas was extracted for gas chromatographic analysis by lowering the pressure, and no O_2_ could be detected. After that, the pressure was raised to 22 MPa so that the gas was fully dissolved in the heavy oil. At the end of the experiment, we reduced the pressure to 1 atmosphere and measured the O_2_ content by gas sampling for chromatographic analysis. The O_2_ content was 1.38 mol %, indicating that this part of O_2_ was dissolved in the oil at 20 MPa and released from the oil when the pressure was reduced from 20 MPa to 1 atmosphere. The 1.38 mol % O_2_ is believed to be the part that did not participate in any chemical reaction. The HTHP experiment indicates that of the initial 5 mol % O_2_, at least 28% (1.38 mol % O_2_) dissolves into the heavy oil and 72% (3.62 mol % O_2_) is consumed by either dissolution or reaction in the oil during the experiment.

After the HTHP reaction experiment, the element analysis technique was used to analyze the elemental compositions of heavy oil before and after the DBFG was injected into the heavy oil. The results show that the contents of hydrogen and nitrogen in the heavy oil slightly reduce due to the volatilization of light components, while the content of oxygen increases, indicating that some oxygen-containing compounds are generated in the oxidation process of heavy oil at 140 °C (see Table 3). It is very interesting to see what the oxidation products could be.

**Table 3:** Comparison of Heavy Oil Sample Elements before and after the HTHP Reaction Experiment^a^

sample	C (mol %)	H (mol %)	O (mol %)	N (mol %) (CHN)	S (mol %)
S1	86.64	11.62	1.5	0.67	0.61
S2	85.85	11.42	2.31	0.64	0.53

a S1 is the primary oil sample, and S2 is the oil sample after the HTHP reaction.

#### 2.3.2 Oxidation Reaction Analysis

**Figure 10:**
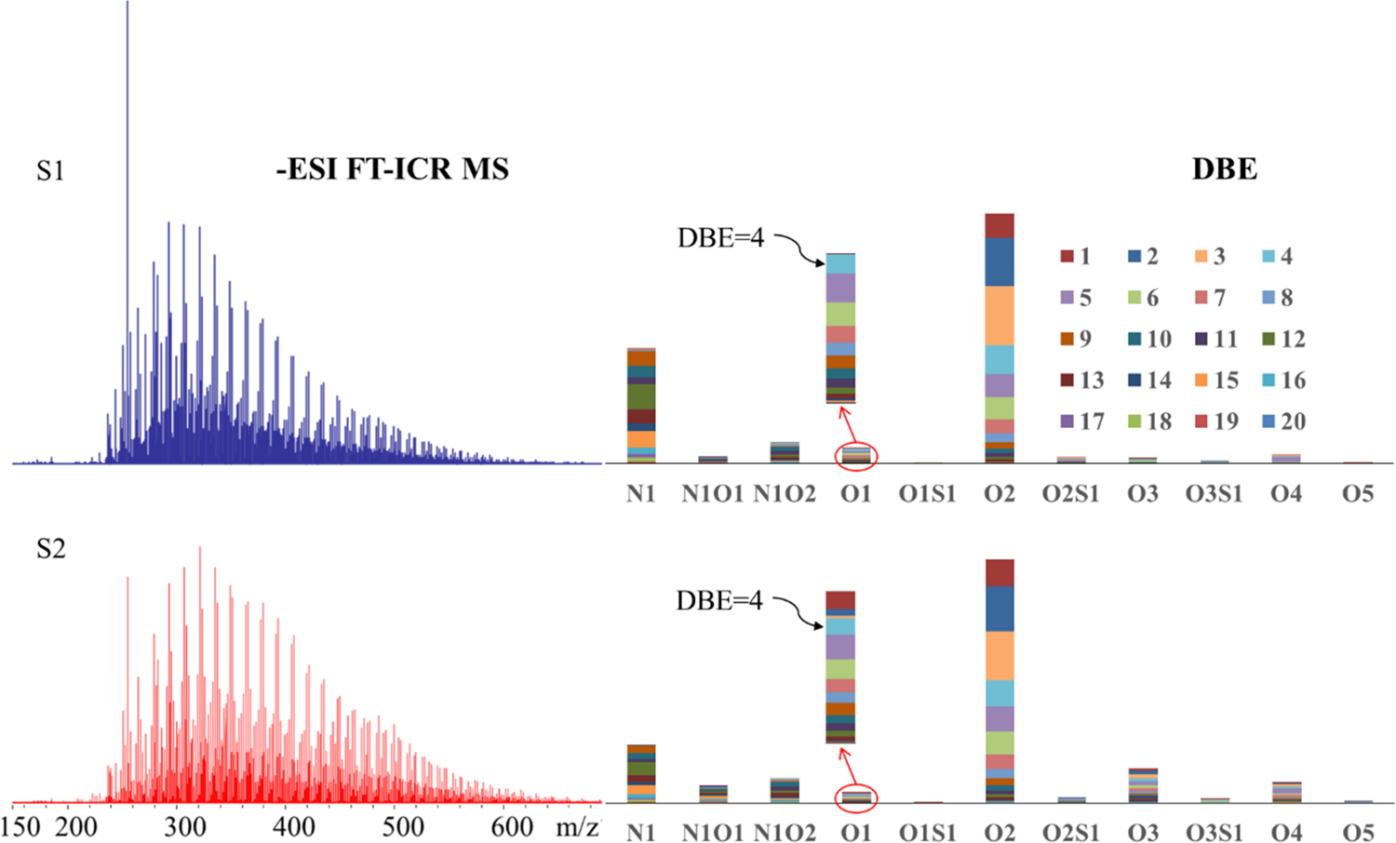
Negative-ion ESI FT-ICR mass spectra and relative abundance of compound classes assigned from the mass spectra of two oils.

Figure 10 shows the full negative-ion ESI FT-ICR mass spectra and the relative abundances of detected classes (N_1_, O_1_, O_2_, O_3_, O_4_, O_5_, N_1_O_1_, N_1_O_2_, and O_2_S_1_ components) of the primary oil sample (S1) and the oil sample after the HTHP reaction (S2). On the basis of common knowledge of electrospray ionization, the N_1_ species, O_1_ species, and O_2_ species mainly correspond to neutral nitrogen compounds, alcohols, and carboxylic acids, respectively.^38^ The N_1_O_1_, N_1_O_2_, O_2_S_1_, O_3_, O_4_, and O_5_ species have different functional groups but at least can be ionized by negative-ion electrospray ionization (ESI). The results indicate that the O_2_ classes constitute the maximum acid portion of the heavy oil sample before and after the HTHP reaction experiment. However, there is obvious difference before and after the HTHP reaction for the O_1_ species, but not for the O_2_ species.

**Figure 11:**
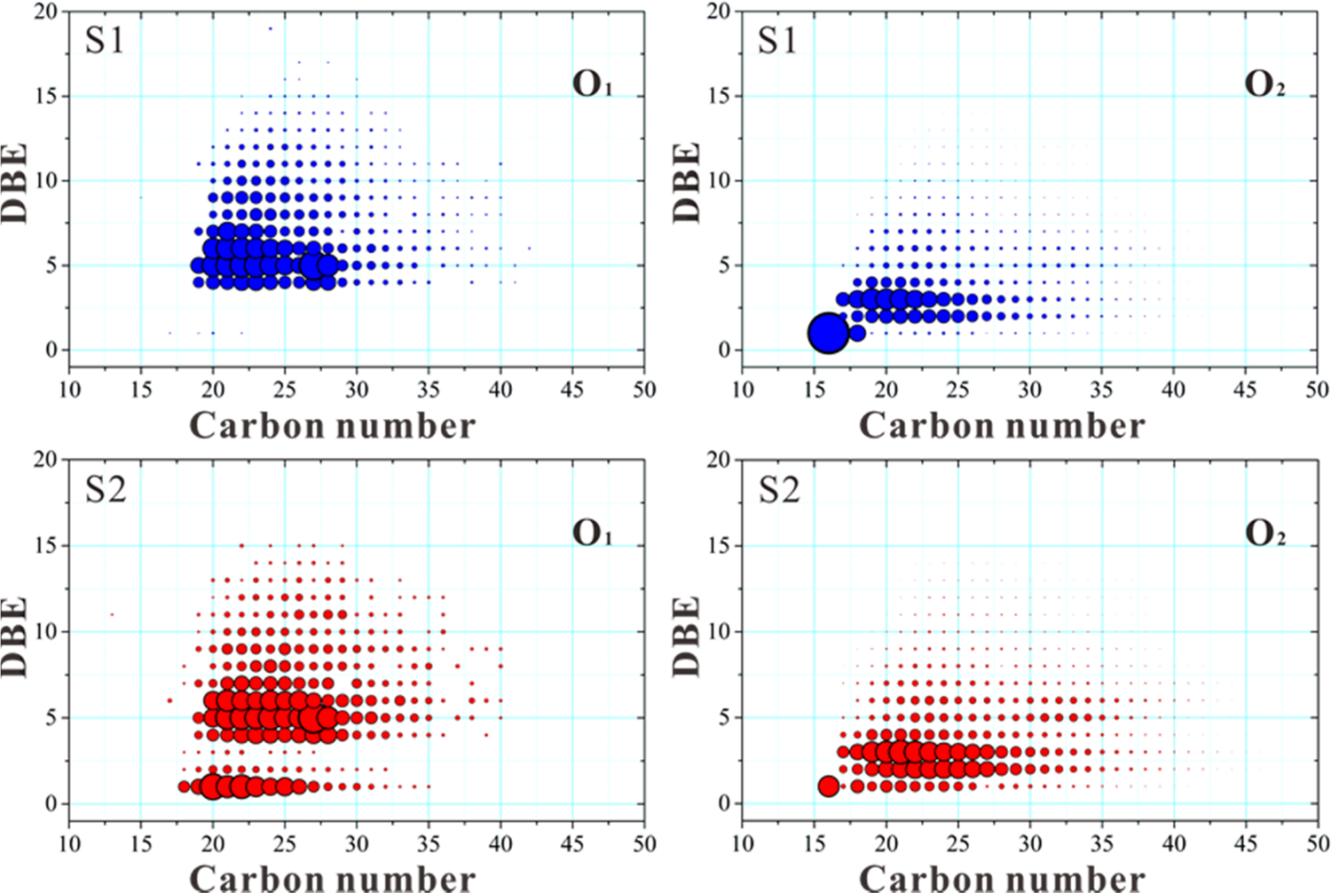
Relative abundance changes with DBE and carbon number of the hydrocarbons.

Figure 11 shows the O_1_ and O_2_ species in two oil samples. The DBE value versus the carbon number obtained by Fourier transform ion cyclotron resonance mass spectrometry (FT-ICR MS) is plotted for comparison. The dot size represents the relative abundance of the component in the heavy oil sample. The larger the dot size, the more the relative abundance of the component. The plots clearly indicate that there is a new O_1_ class formed in the primary oil (sample S1) after the HTHP reaction, analyzed by negative-ion ESI. Abundant O_1_ species with carbon number in the range of 17–35 and DBE values in the range of 1–3 imply that the primary oil sample is oxidized to form alcohols. However, there is no obvious difference in O_2_ species of the two oil samples before and after the HTHP reaction. Therefore, it can be inferred that the oxidation reaction indeed occurred between the heavy oil and DBFG at 140 °C and the oxidation product is alcohols, not acids.

The result reveals that the oxidation reaction indeed occurs after the contact between the heavy oil and DBFG at the HTHP condition, although the reaction is not violent. After the heavy oil is oxidized, alcohols are generated. It should be noted that the HTHP mentioned here refers to the DBFG-assisted steam stimulation for the heavy oil EOR method, in which the temperature can reach 100–300 °C and the pressure can reach 10–20 MPa. It is very different from the in situ combustion method, where oxidation reactions occur more violently since the maximum temperature can reach 600–800 °C.^7,9,39−41^

In summary, based on the results of the HTHP experiment and the high-resolution FT-ICR MS, we can see that under the DBFG-assisted HTHP condition, the oxidation reaction occurs between the heavy oil and the O_2_ in DBFG, resulting in a change in the composition of the heavy oil. The reaction also could be the cause of more DBFG being dissolved in the heavy oil, leading to the increased solubility shown in Figure 7, and the volume may swell up, leading to the increased swelling factor shown in Figure 8. In general, the small amount of nitrogen oxides, sulfides, and particulate matter in DBFG will not have a significant impact on the properties and composition of heavy oil, so it is unnecessary to remove these components in DBFG in the heavy oil production process, which can not only reduce the emissions of greenhouse gas and pollutants but also save costs and enhance the heavy oil recovery.

### 2.4 Application Prospect of Real Oil Fields

Injecting flue gas into oil reservoirs to enhance oil recovery has been studied for nearly 60 years.^14^ Coinjection of steam and flue gas directly from the steam generator to carry out CSS and SAGD has been proven successful by pilot tests.^17,18,42^ This technology has the potential to reduce steam heat loss, promote the development of the steam chamber, reduce the cost of injection substantially, provide a means to sequester some of the exhaust gas from steam generation, and capture CO_2_ in the oil reservoir.^17^

The CO_2_ sequestration capacity of the oil reservoir is affected by multiple factors, such as reservoir properties, reservoir sealing, burial depth, reservoir pressure, porosity, relative mobility ratio of the CO_2_ and oil, gravity segregation, reservoir heterogeneity, and the volume of invading water, so the theoretical CO_2_ sequestration capacity of the ideal state cannot be achieved.^43^ According to 45 miscible CO_2_ injection projects in North America, approximately 40% of the originally injected CO_2_ is produced, which means that about 60% of the injected CO_2_ is captured by the oil reservoir, while nearly 33–50% of the injected CO_2_ is captured by the oil reservoir in China.^44,45^ Thus, according to above data, we estimate that about half of the CO_2_ in DBFG (9% CO_2_) will be really “buried” for long-term storage.

Therefore, enhanced oil recovery using DBFG has broad application prospects in real oil fields.

## 3 Conclusions

In this paper, with the aim of directly injecting high-temperature DBFG with all components, i.e., N_2_, CO_2_, O_2_, and small amounts of nitrogen oxides, sulfur compounds, and particulate matter, into heavy oil during the EOR process, we carried out a series of experiments to understand the influence of DBFG on the properties and composition of heavy oil.

The fly ash particulate matter from burning heavy oil shows two well distinct structures: cenospheres and nanoscale dense particles deposited on the surface of cenospheres. The main PSD of the cenosphere particulate matter ranges from 10 to 40 μm. The major elements of cenospheres comprise carbon, oxygen, and sulfur, while the dense particles deposited on larger cenospheres comprise oxygen, carbon, silicon, aluminum, and ferrum. When the concentration of particulate matter in the heavy oil is less than 0.5 wt %, the effect of particulate matter on the viscosity of heavy oil can be almost ignored.

PVT studies show that the DBFG dissolved in heavy oil can reduce the oil viscosity, increase the flow capability, and make the heavy oil volume swell. Under a constant pressure, the solubility and swelling factor increase as the temperature increases, especially when the pressure is above 13 MPa.

The HTHP experimental results showed that oxidation occurred between heavy oil and DBFG at 140 °C, the compositions of heavy oil changed, and the oxidation product was alcohols. More DBFG may be dissolved in heavy oil, and the volume may swell up. The small amount of nitrogen oxides, sulfides, and particulate matter in DBFG will not have a significant impact on the properties and composition of the heavy oil.

The findings of this study indicating the beneficial effect of DBFG on the viscosity and swelling factor are very encouraging because it is expected that DBFG can be directly injected into heavy oil, not only for EOR but also for reducing pollutants, as well as saving costs. It is of utmost importance to find a way to reduce the emissions of greenhouse gas CO_2_ that will help China reach the ultimate goal for carbon peak before 2030 and carbon neutral by 2060.

## 4 Experimental Section

### 4.1 Materials

The heavy oil used in the experiment was sampled from the Neogene Guantao formation (Ng) reservoir in the Shengli Oilfield (China). The formation temperature and pressure are 60 °C and 10 MPa. Its basic properties are shown in Table 4.

**Table 4:** Properties of the Heavy Oil Used in the Experiments^a^

property	unit	value
density at 20 °C	g/cm^3^	0.9632
viscosity at 60 °C	mPa.s	805.63
saturate content	wt %	39.83
aromatic content	wt %	25.70
resin content	wt %	22.27
asphaltene content	wt %	6.85

a Analysis was conducted at Bohai Central Lab, Engineering Technology Branch, CNOOC Energy Development Co. Ltd., (Tianjin, China).

The DBFG from the steam generator boiler at the Shengli Oilfield was collected, and its composition was analyzed. Since the DBFG from the steam generator boiler was not convenient to be compressed and transported to the laboratory, the DBFG used in the experiments was prepared in the laboratory based on the DBFG compositions from the steam generator boiler, which was composed of 9 mol % CO_2_, 5 mol % O_2_, 145 ppm NO_2_, 768 ppm SO_2_, and N_2_ as the equilibrium gas. The purity of each gas (Tianling Inc., China) used in this work was 99.999%. The DBFG was stored in a 10 L cylinder at a pressure 12 MPa.

Particulate matter in this study was sampled from the steam generator boiler burning heavy oil in the Shengli Oilfield. The particulate matter was sifted with 60-, 150-, and 300-mesh sieves and allowed to stand for a period of time for the precipitation of micro-/nano-size particles.

### 4.2 Particulate Matter Imaging

The particulate matter was imaged using a field emission scanning electron microscope (SEM, Zeiss Merlin) with a spatial resolution of 0.8 nm. All samples were examined using a secondary electron detector for morphological analysis and particle size distribution statistics at an accelerating voltage of 1.5 kV. SEM in conjunction with an energy-dispersive spectrometer (EDS) was used to measure various elements in the particulate matter at an accelerating voltage of 15 kV.

### 4.3 Viscosity Measurement

A MARS III rotation rheometer (HAAKE, Germany) was used to measure the viscosity of the heavy oil with the particulate matter. A special adapter was installed in this apparatus, with a temperature range from room temperature to 300 °C and viscosity measurements ranging from 1 × 10^–3^ to 1 × 10^12^ mPa.s. First, we prepared two 50 mL beakers, by cleaning, drying, and weighing them. Second, we poured the dehydrated dead oil into the beaker and weighed it separately. Then, we added 0.5 wt % particulate matter to one beaker and weighed it after stirring well. Last, we measured the viscosity by the MARS III rotation rheometer.

### 4.4 PVT Equipment and Experimental Procedures

**Figure 12:**
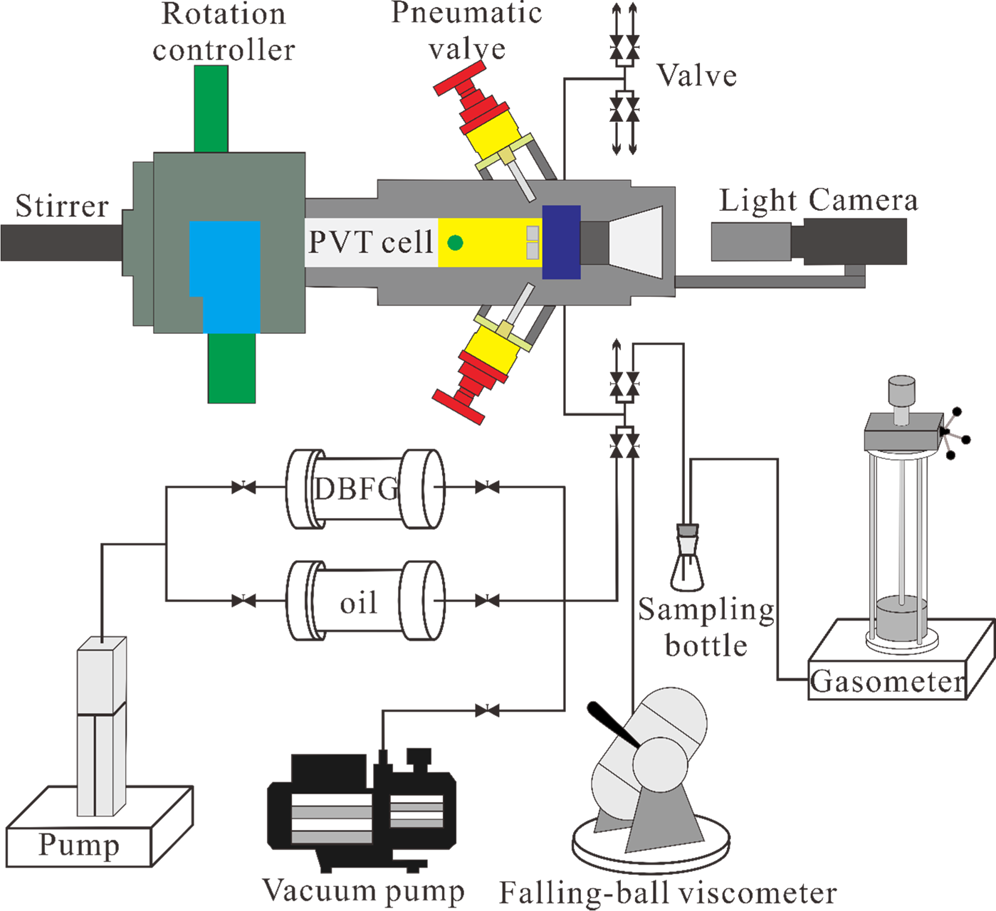
Schematic diagram of the PVT experiment system.

The PVT equipment used in this experiment was a PVT 400/1000FV system (ST, France) (Figure 12) for understanding the solubility of DBFG and its effect on swelling, viscosity reduction, and density of the heavy oil. The total volume of the PVT cell was 400 mL, with a pressure range from atmospheric pressure to 100 MPa, temperature range from room temperature to 200 °C, and temperature accuracy of the cell being ± 0.01 °C.

The falling-ball viscometer was used to measure the viscosity during the PVT experiment, with a pressure range from atmospheric pressure to 100 MPa, temperature range from room temperature to 200 °C, viscosity range of 0.1–10 000 mPa.s, and temperature accuracy of ± 0.1 °C.

The PVT experimental procedures were composed of six steps. First of all, the PVT cell was cleaned with kerosene, blown dry with nitrogen, and vacuumed. Second, 0.5 wt % solid particulate matter was added to a certain volume of dehydrated dead oil; this was mainly because the solid particulate matter cannot be injected into the heavy oil together with DBFG, and then dead oil and DBFG were injected into the PVT cell at desired temperatures of 60, 100, and 140 °C. Third, the pressure of the mixture of DBFG and heavy oil was pumped to 30 MPa, with the stirrer kept rotating and the PVT cell swinging up and down, which ensured that all of the DBFG was dissolved in the heavy oil. Fourth, the pressure of the mixture was decreased to 20 MPa; after equilibration, the free gas at the top of the cell was removed, while the pressure was maintained constant by raising the piston. The pressure in the cell was then increased, and the mixture of heavy oil and DBFG was mixed for about 6 h to obtain a homogenized single phase sample. Fifth, a sample of the heavy oil saturated DBFG was collected in the sampling bottle and the gasometer for the determination of density, solubility, and swelling factor. After that, a certain volume of heavy oil saturated with DBFG was injected into the falling-ball viscometer with the pressure greater than the saturation pressure, which was taken to prevent degassing. The viscosity of the live oil was measured 5 times until the readings did not vary by more than 5%, and the averaged value was adopted. At last, the pressure of the mixture was decreased, and the pressures and volumes of the mixture were recorded and plotted in a figure. The saturation pressure could be determined by the inflection point in a pressure–volume curve. The same process was repeated at 16, 12, and 8 MPa.

### 4.5 HTHP Reaction Apparatus and Procedures

**Figure 13:**
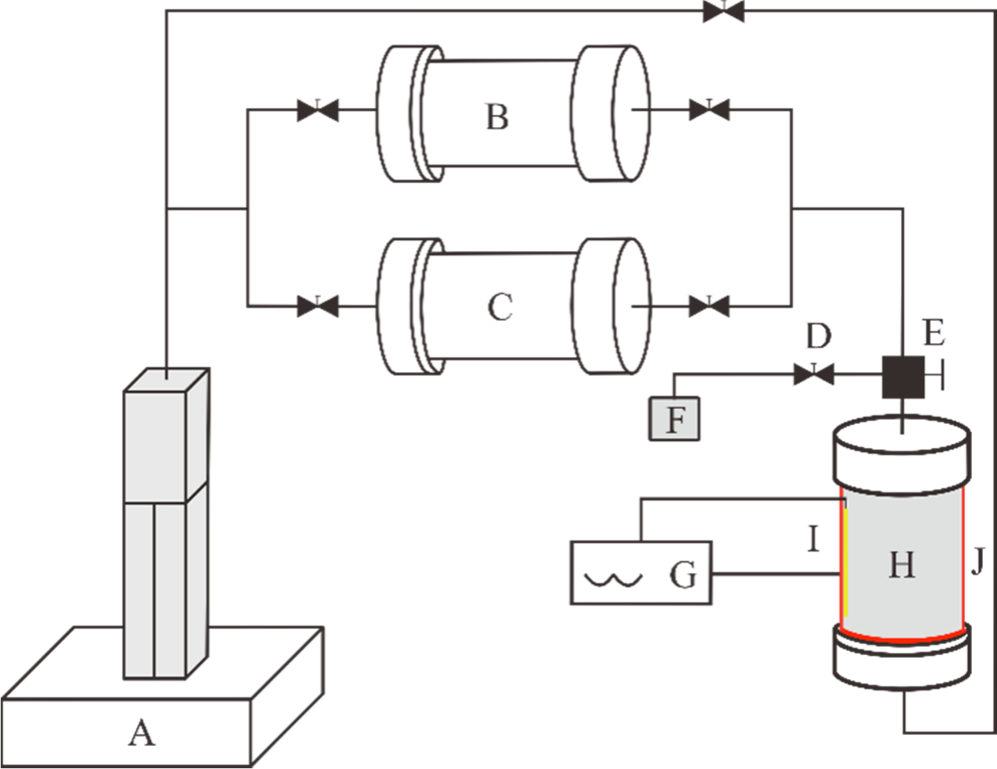
Schematic diagram of the HTHP reaction apparatus. (A) pump, (B) transfer cylinder with heavy oil, (C) transfer cylinder with DBFG, (D) valve, (E) three-way valve, (F) gas sampling bag, (G) temperature controller, (H) reaction still, (I) thermocouple, and (J) heater band.

To understand the influence of O_2_, nitrogen oxides, and sulfur compounds in DBFG on heavy oil compositions under HTHP conditions, we performed experiments using an HTHP reaction apparatus as shown in Figure 13. The HTHP experimental apparatus includes a pump, a transfer cylinder with heavy oil and DBFG, a valve, a three-way valve, a sampling bottle, a temperature control instrument, a reaction still, a thermocouple, a high-pressure pipeline, and a heater band. The total volume of the HTHP reaction still was 100 mL, with a pressure range from 1 atmosphere to 40 MPa and a temperature range from room temperature to 200 °C.

First of all, all of the instruments were cleaned and the air tightness of the HTHP experimental apparatus was checked to make sure no leakage happened. Dehydration dead oil (50 mL) was injected into the reaction still at room temperature. Second, reaction was heated up to the desired temperature of 140 °C. After 5 h, 50 mL (room temperature, 10 MPa) of DBFG was injected into the reaction still and the pressure was increased to 30 MPa for real-time measurement of the experimental pressure until a constant pressure was achieved. After the pressure did not change, it was allowed to keep for 24 h to allow the DBFG and heavy oil to fully react. Third, we reduced the pressure to 20 MPa through adjusting the pump and discharging the excess gas and kept the pressure constant for 40 h. At the end of the experiment, we reduced the pressure to 1 atmosphere and stopped heating until the reaction still was cooled to room temperature. The gas was analyzed by gas chromatography, and the oil sample was analyzed before and after the reaction by an element analysis instrument and a high-resolution mass spectrometer.

### 4.6 Element Analysis Instrument

Element analysis of heavy oil samples was conducted using a Vario EL cube and a rapid OXY cube, equipped with a thermal conductivity detector (TCD, Elementar Analysensysteme GmbH, Germany), with the detection limit of each element being 0.01 wt %.

### 4.7 High-Resolution Mass Spectrometer

In recent years, the application of FT-ICR MS to analyze the composition of heteroatomic compounds in heavy oils has seen a breakthrough.^46−48^ Thus, FT-ICR MS analysis was carried out to characterize the compounds. A Bruker Apex-Ultra FT-ICR MS equipped with a 9.4 T superconducting magnet was used in the ultrahigh-resolution MS experiment. Positive ion-atmospheric pressure photoionization (APPI) and negative electrospray ionization (−ESI) sources were the most commonly used on FT-ICR MS to analyze compounds. More details of the equipment and operating procedure can be found in previous works.^49,50^
